# The Influence of Social Media in Promoting Knowledge Acquisition and Pathology Excellence in Nigeria

**DOI:** 10.3389/fmed.2022.906950

**Published:** 2022-06-03

**Authors:** Olaleke Oluwasegun Folaranmi, Kehinde Muibat Ibiyeye, Olabode Ali Odetunde, Darcy A. Kerr

**Affiliations:** ^1^Department of Anatomic Pathology, University of Ilorin Teaching Hospital, Ilorin, Nigeria; ^2^Department of Pathology and Laboratory Medicine, Dartmouth-Hitchcock Medical Center, Lebanon, NH, United States; ^3^Department of Pathology, Geisel School of Medicine at Dartmouth, Hanover, NH, United States

**Keywords:** social media, pathology, medical education, professional development, Nigeria, Africa

## Abstract

The use of social media has evolved from platforms designed primarily for social connection and news sharing to include vibrant virtual academic environments. These platforms allow pathologists from across the globe to interact, exchange knowledge, and collaborate. Pathology in Nigeria, as in much of Africa, faces severe knowledge and practice gaps, with a lack of supporting modern laboratory infrastructure. Social media represents a potentially highly valuable avenue to help address some of these deficiencies. In this Perspective piece, we highlight our experience with the increasing role of social media in providing quality medical education in pathology globally, with an emphasis on how it bridges many of these gaps in Nigeria. Social media sites serve as sources of readily accessible, free, high-quality information to pathologists and trainees through academic discussions, quizzes, journal clubs, and informal consultations. They also provide opportunities for professional networking and research collaborations. Despite the availability and wide reach of these platforms, social media as a tool for advancement of knowledge in pathology is still undersubscribed in this part of the world. Improving awareness of and support for these tools will ideally help mitigate some of the challenges of practicing pathology in low and middle-income settings.

## Introduction

Beyond facilitating connections between individuals, social media use now significantly impacts political, commercial, and scientific circles. People of different ages, backgrounds, and geography use social media for a range of purposes within medicine. Pathologists engage in rich virtual academic environments that foster interaction, knowledge sharing, and collaboration, while not being limited by typical geographic, social, or cultural boundaries (1). An advantage of social media is the opportunity to curate information based on topics/areas of interest (2).

A decade ago, it may have been difficult to imagine social media as a powerful tool in medicine given the ethical and legal concerns surrounding patient privacy (3, 4). Many of these issues have now been addressed by published articles that provide guidelines for the professional use of social media by physicians, improving the adoptability of social media as a legitimate channel for discussing medical cases (1, 5–7). These sites have become hubs for a growing and enthusiastic worldwide audience to disseminate medical knowledge and foster professional networking (8).

The training and practice of pathology in many low- and middle-income countries, including Nigeria and much of Africa, is many decades behind what is currently obtainable in high-income countries. Social media can be a valuable tool to help ameliorate some of these deficiencies. Herein, we present our perspectives on the current role social media plays in providing a high-quality adjunct to formal medical education in Nigeria, contextualized within current social media use in pathology. Further, we consider relevant limitations and potential future directions.

## Pathology Practice in Africa

Pathology services in most of Africa today lag considerably behind practice in high-income countries. This is principally due to limited resources with resultant poor healthcare financing (9). The budgetary allocation to the health sector in Nigeria for 2022 is almost 30% higher than it was for the previous year but still <5% of the total budget. This is far from the commitment made by leaders of the African Union in 2001 to allocate at least 15% of their annual budget to improve healthcare in Africa (10). Practicing in an environment that lacks requisite infrastructure greatly impairs routine services and the training of laboratory professionals, and it almost totally excludes the ability to conduct meaningful research. These inadequacies have severely impeded the fight against cancer (11). With prevailing low income and limited health insurance coverage, only a small proportion of the population can afford healthcare. As a result, most pathology laboratories process few samples, with the majority receiving <3,000 tissue samples annually (12). Low case volume and limited availability of ancillary techniques severely impair the proficiency of pathologists in this region. Indeed, in Nigeria, like nearly all of Africa, there is no current capacity for subspecialty training. Most training institutions do not have subscription access to journals or a repository of current literature. To illustrate, in one of the author's experience (O.O.F.), it is a struggle to get access to at least seven in 10 journals of interest. All these, coupled with low numbers of laboratory professionals have made pathologists almost invisible in this part of the world (12, 13).

## Pathology and Social Media

The most commonly utilized social media platforms in pathology include Facebook, YouTube, Instagram, and Twitter. For an excellent review summarizing the use of these platforms in pathology, authors would refer interested readers to a recent article by Deeken et al. (14). In brief, Facebook has the most robust, global network and reach. Some pathologists share educational material in open professional accounts. Facebook groups allow interested parties to unite over a particular topic, even connecting pathologists and patients, and crowdsourcing images of rare entities for textbooks. Facebook allows posting of educational videos and, in particular, Facebook Live allows one to host interactive microscopy sessions. YouTube focuses on video sharing, widely accessible lectures, and case discussions, and it is the most popular site for these purposes. This platform facilitates access to the types of educational materials that are commonly sold for profit or Continuing Medical Education credits. It allows for a very wide distribution of materials, and videos may garner 50,000–100,000 views in one to several years. Instagram, a platform where images are central, is well-suited to pathology for its visual nature but is suboptimal for fostering conversations. Unlike other sites, one cannot share links or articles, impeding robust discussion (14). Twitter is a US-based microblogging site notable for its wide reach, visual nature, “open” network, and ability to encourage discourse around educational cases through creating and sharing “threads,” where relevant information is embedded and linked. The ability to crowdsource cases and develop research collaborations are also strengths of Twitter (15). For the authors, Twitter is the platform with which we have the most professional experience, and we believe it to be uniquely suited to helping promote knowledge acquisition and pathology excellence. Therefore, we will largely focus on this platform for the remainder of this piece, while recognizing many of the elements are also applicable to other social media platforms.

Launched as “Twttr” in March 2007 as a free instant messaging service, today Twitter is a popular source of information for a wide variety of industries (16). In the last few years, a large number of pathology Twitter accounts have proliferated, including accounts for individuals, institutions and organizations/associations. In 2019, a list kept by Jerad Gardner, et al. (1) documented more than 4,700 active pathology-related Twitter accounts. Two years later, this number almost doubled (17). Pathology-related posts can be readily searched for by using the hashtag #PathTwitter, with many other specific tags for organ systems or subspecialties (e.g., #Dermpath for Dermatopathology and #GIpath for Gastrointestinal Pathology) or branches of pathology (e.g., #Cytopath for Cytopathology and #ForensicPath for Forensic Pathology) (7, 14, 18). See Appendix for Supplementary Information.

## The Impact of Twitter on Pathology Practice in Nigeria: Our Perspective

**Figure 1:**
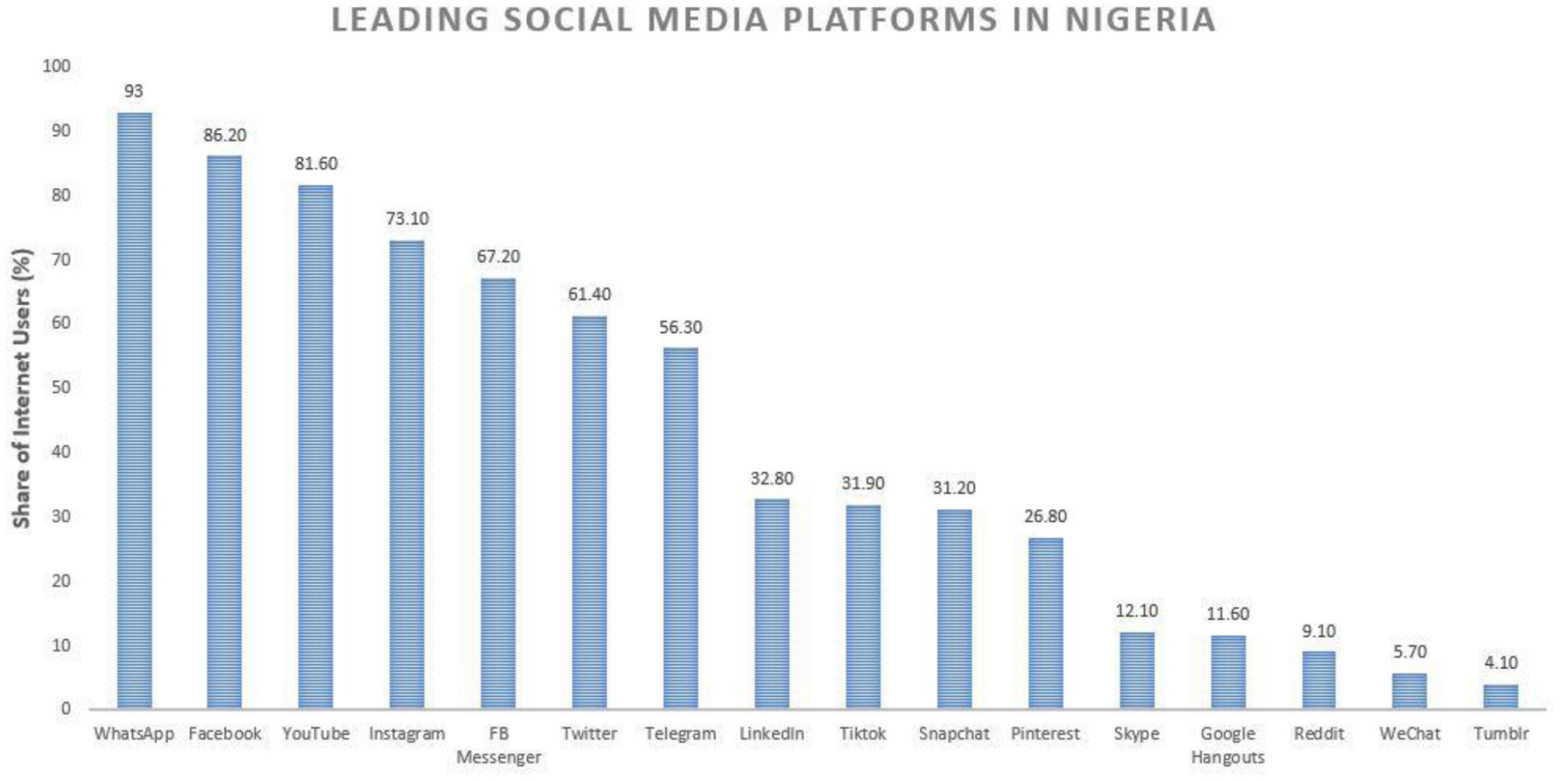
A graph highlighting the popularity of various social media apps/sites commonly used in Nigeria. The information is based on survey data from internet users aged 16–64 years, as of the third quarter of 2020 (https://www.statista.com/statistics/1176101/leading-social-media-platforms-nigeria/) .

Very few studies have been done to assess the extent to which African pathologists use social media professionally (19–21). Many pathologists in this part of the world, as elsewhere, began using social media accounts primarily for personal purposes, most commonly WhatsApp and Facebook (Figure 1). Over the years, these platforms have increasingly been used to communicate with colleagues in the same region and sometimes seek consultations by sending images of difficult cases, although with limited use (20).

There is no official data on the exact number of pathologists practicing in Nigeria. About 2 years ago, the College of Nigerian Pathologists estimated that there were about 500 pathologists practicing in the country, a nation of over 200 million people (22). Most of these pathologists have no active presence on Twitter. Occasionally, a few pathology residents and consultants can be spotted engaging, rebroadcasting (retweeting) and commenting on pathology-related posts. Very few maintain active participation, posting pathology images to teach or to seek opinions in difficult cases. Therefore, the following discussion is based on the experience of these few, including two of the authors (O.O.F. and O.A.O).

In Nigeria, Twitter has been a force of positive impact in our pathology practice by providing: (1) a rich source of medical education; (2) a platform for case consultation; (3) a niche for professional networking and mentorship; and (4) a tool for research collaboration.

### Twitter as a Source of High-Quality, Free Education in Pathology

Twitter is an excellent source of high-quality pathology material contributed by all cadres of pathology lovers, ranging from medical students on pathology electives to residents, fellows and attendings. Pathologists from all career stages (including post-retirement) and practice settings share their insights and experiences (1).

The topics discussed cover diverse aspects of pathology: gross and autopsy techniques, microscopic findings, specimen photography tips, artifacts, and diagnostic pearls and pitfalls. Discussions also span all pathology subspecialties. These interactions have provided us ample exposure to content areas that are missing in the training and practice of pathology in Nigeria. To put this in context, a 2019 survey assessing pathology services in the country revealed that only a quarter of the 16 participating institutions had immunohistochemistry. Only two had the capacity for other ancillary techniques such as (frozen sections, *in-situ* hybridization and polymerase chain reaction) (12). Therefore, daily Twitter exposure to a variety of cases with robust discussions on ancillary techniques has been of great educational value.

**Figure 2:**
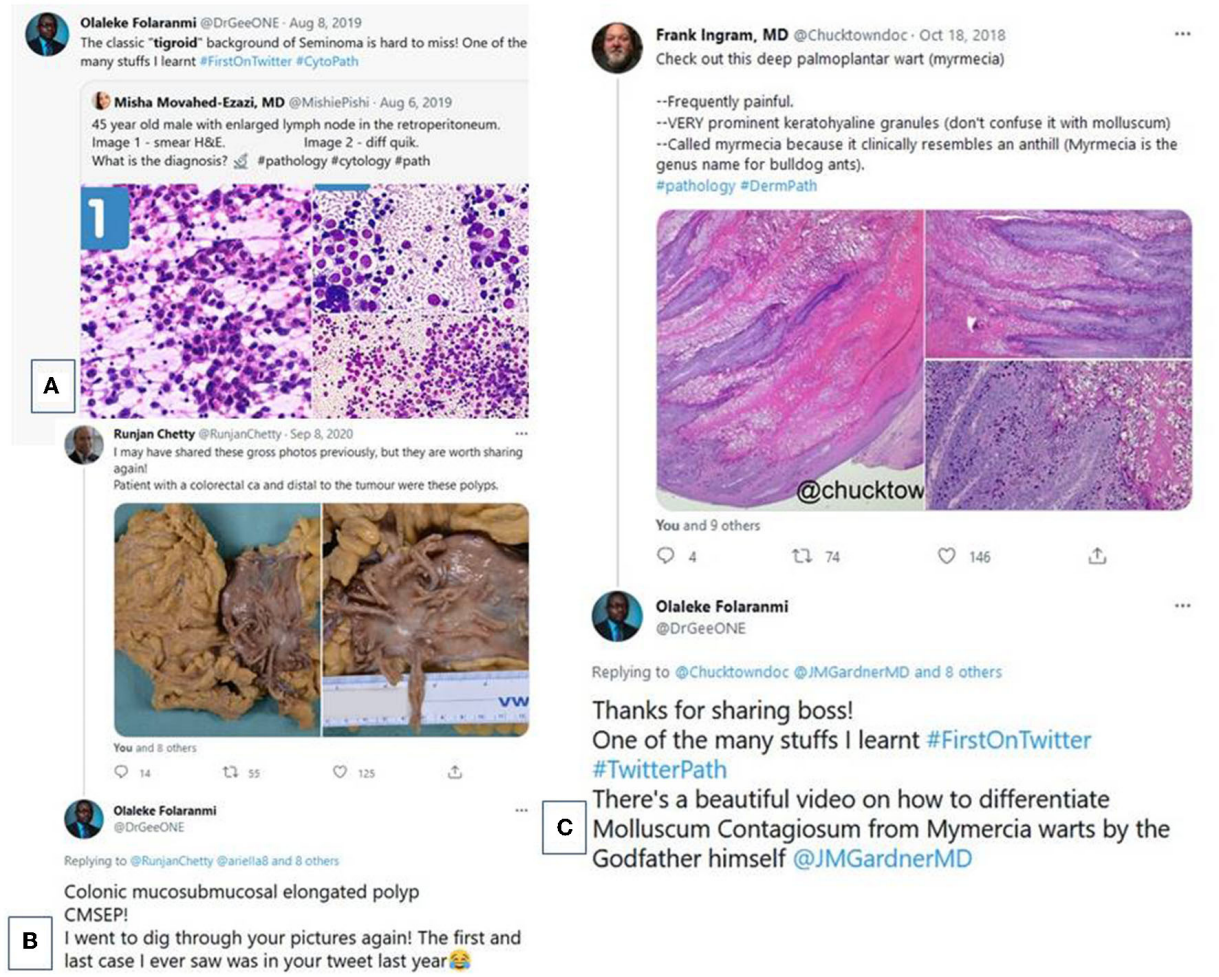
Interactions with educative tweets. (**A)** A quoted tweet from Misha Movahed-Ezazi, MD (@MishiePishi) by one of the authors amplifying one of the many valuable lessons in cytopathology that was learnt first on Twitter: the “tigroid” background of seminoma. (**B)** A tweet by Frank Ingram, MD (@Chucktowndoc) highlighting the salient facts in distinguishing between Myrmecia warts and Molluscum based on morphology. (**C)** A tweet by Runjan Chetty, FRCPC, FRCPath (@RunjanChetty) showcasing an extremely rare lesion ‘colonic mucosubmucosal elongated polyp.'

This social media platform particularly shines as a source of up-to-date information, which is essential for all pathologists but more difficult for pathologists working in low-resource settings to access. As a resident and fellow, one of the authors (O.O.F.) and colleagues in other centers can recollect a variety of cases that were hitherto unknown to them until they were encountered on Twitter. Some valuable cases range from seemingly simple ones, such as recognizing the classic “tigroid” background of seminoma on cytology smears (Figure 2A) or appreciating the differences between Molluscum contagiosum and Myrmecia warts (Figure 2B) to seeing heretofore unheard-of lesions such as the rare colonic mucosubmucosal elongated polyp (Figure 2C) (23–30). Some of these cases would later cross our trays not long after we saw them on social media (26, 29). A testament to the immense value of this educational content, O.O.F. stated: “I have learned more pathology from interactions with pathologists and residents on Twitter and Facebook than from physical interactions with people in all my years of training” in an interview, “Education Beyond Borders” by media specialist, Dustin Johnston (31).

### Twitter as a Platform for Case Consultation

Most interactions on #PathTwitter are focused on learning through mini case presentations, Twitter-based tutorials (Tweetorials), and quizzes. However, Twitter also provides an avenue to share difficult cases and seek opinions and guidance from those more experienced in certain subspecialties. Almost all Nigerian pathologists are generalists by training and in practice with few having formal subspecialized training. The interest of one of the authors (O.O.F.) in the field of dermatopathology was spawned when a specialist dermatology clinic was set up in his center. There was no known dermatopathologist nearby for the treating physicians to consult, and very few pathologists had an interest in this field as it was perceived to be difficult. Facebook groups (especially the McKee Derm group) and Twitter were the only readily accessible platforms to learn dermatopathology and to share images for guidance. Such unofficial consultations come with an unwritten caveat: the final diagnosis lies with the pathologist who has the glass slides and the clinical history (32). Nevertheless, having access to experts in a particular field who volunteer helpful comments and share opinions on how to approach difficult cases has brought more confidence to our practice (33). The few Nigerian pathologists who are active on Twitter continue to leverage this great opportunity to learn more, bringing the best care available from across the world to patients in Nigeria (33–39).

Technological advancements have greatly improved the way pathologists share cases; glass slides can now be scanned and rendered as high-definition digital images [whole-slide imaging (WSI)]. WSI enables access to the “full picture” of the tissue on the slide, allowing for a more comprehensive case review (40). The adoption of these images is growing on Twitter. Leading here is the “Knowledge in Knowledge Out” [KiKo, (@kiko4docs)] platform founded by Jonhan Ho, MD (@forthejon) to enable physicians share digital images for consultation and teaching purposes.

### Twitter as a Niche for Professional Networking and Mentorship

In the traditional sense, most professional networking arises from interactions at physical meetings such as international conferences. Travel grants for people practicing in resource-constrained environments are limited and have become highly competitive. However, in the last few years of consistent interaction with pathologist colleagues on Twitter, we have seen increased followers and a growing interest from around the world in the cases we present. Additionally, these regular interactions have grown into professional relationships leading to invitations to participate in academic activities such as virtual seminars. In 2020, one of the authors (O.O.F.) was selected to be on the team of international screening judges that curate and vote for tweets nominated for PathTweetAward. This Twitter-based, crowd-funded initiative founded by Sanjay Mukhopadhyay, MD (@smlungpathguy) and Amy Deeken, MD (@AmyHDeekenMD) in April 2018 seeks to promote educational Twitter posts by awarding prizes in open and trainee categories (18). Furthermore, we have seen the effects of Twitter promoting development of “real life” mentors. These mentorship pairings have resulted in increased pathology clinical experience, research publications, and post-graduate training opportunities for Nigerian-trained medical graduates.

The benefits from these interactions and networking have been tremendous: colleagues providing mentorship and inspiring excellence in one's practice, donations of personal equipment, books, and more. Engagement on Twitter has drawn more attention to the knowledge and infrastructure gaps that exist between high-income countries and low- or middle-income areas such as Nigeria (31).

### Twitter as a Tool for Research Collaboration

The first published international collaboration generated from and facilitated by a social media post originated in a tweet from Lara Pijuan, MD (@lara_pijuan), a cytopathologist from Spain, who described iatrogenic displacement of cartilage fragments into mediastinal nodes from endobronchial ultrasound-guided fine needle aspiration procedures (41). When the finding was later recognized by another pathologist, it spurred an international collaboration resulting in multiple presentations and publications, including an original research paper (15) as well as a separate manuscript outlining the process of generating and completing a pathology research study through social media (42). The first collaborative project involving Nigerian pathologists through Twitter occurred in 2020. Andrew Schaumberg, PhD (@schaumberg_a), a post-doctoral researcher working on medical machine learning and computational pathology, enlisted the help of pathologists on Twitter for a machine learning project. The project used histologic images from 25 pathologists residing in 13 countries and included two Nigerian pathologists (43, 44). These projects highlight the far-reaching and growing potential for Twitter in promoting global pathology collaborations.

## Challenges With Twitter Adoption in Nigeria

Many pathologists in Nigeria have yet to discover the educational benefits of social media platforms such as Twitter. There are a few reasons why this may be so:

### Lack of Awareness

In Nigeria, WhatsApp and Facebook are the top two platforms that are widely used, whereas Twitter ranks 6th (45). This suggests that many physicians may actually have little information on the use of this platform for beneficial academic activities. Our interactions with colleagues further reflect that many Nigerian pathologists do not know that Twitter serves as a hub for medical education and scientific information.

### Poor Communication and Infrastructure

Access to internet is still a luxury in Nigeria due to many reasons including: supporting infrastructure deficit, unstable electricity, and the high cost of maintaining equipment, among many others. The number of people with access to the internet is 141 million subscribers, but broadband services have penetrated <50% of these subscribers. These factors have translated to poor internet services and high cost of access (46–48). Furthermore, in order to obtain and share images on social media, investment in a camera and related equipment is necessary and may come at a personal financial cost to the pathologist (31).

### Political Unrest

In recent years, more African countries have shut down internet access with most targeting social media sites during political unrest and elections. Since 2015, about 66 countries have restricted access to social media at one point in time, and African countries account for almost half of these (49). In 2021 alone, five African countries had restricted access to internet and social media due to political turbulence. Nigeria announced the suspension of Twitter operations on June 4, 2021 after a week of tense political atmosphere (49–51). This ban was in effect for 222 days, ending at midnight on January 13th, 2022 (52). These restrictions further impair access to information and educational content in a region already struggling with scarce resources.

## Social Media for Pathologists in High-Income Countries

The use of social media for professional purposes aligns with the goals of many in high-income countries as well, as it has been documented to positively correlate with traditional measures of gauging academic success and productivity. These include garnering increased recognition and citation of published research works and fostering research collaborations. Indeed, social media impact metrics are being incorporated into promotion and tenure criteria (14).

Beyond these traditional measures of academic success, it is recognized that pathologists, like other physicians, frequently have a desire to teach and engage in outreach. Particularly as the global burden of cancer is rising in prominence and global health initiatives reflect this focus on improving cancer care, (53) the role of pathologists is becoming increasingly more relevant. Pathologists are engaged in global health in a range of manners, including but not limited to engaging in more traditional “on the ground” capacity-building projects to virtual education sessions like the Africa Calls teleconferences that have emphasized camaraderie and educational exchange for 20 years, and virtual global tumor boards (54). Indeed, one of the authors (D.A.K.) has an established interest in helping to develop pathology services in low- and middle-income countries, having engaged in several of the above initiatives and projects in Africa (55). The potential to tap into these opportunities on a small scale in spare moments over an extended period of time has emerged for this author as one of the more compelling reasons to engage in Twitter professionally. Similarly, reflecting on interactions with pathology-interested medical students and pathology trainees reveals an interest in global health to be relatively common, (56) supporting the idea that there is a pathway for interested parties to promote and develop mutually beneficial global health initiatives, including those that leverage social media. The potential for social media and traditional in-person capacity-building endeavors to synergistically build on one another's progress should be thoughtfully considered as initiatives are envisioned and deployed.

## Discussion

Nigeria, and Africa as a whole, faces a number of distinct challenges and gaps in the delivery of quality pathology services. Many of the problems in this region stem from poor economic investment in the healthcare sector, an issue that requires wider economic, infrastructure, and political solutions. However, thoughtful professional social media use has the potential to help ameliorate some of these deficiencies, including improving education and diagnostic pathology services, as well as creating mentorship opportunities and research collaborations.

Pathologists, especially those practicing in low-income countries or in remote communities benefit from insights provided by experts when they share challenging cases on these platforms. Although it is difficult to quantify the impact of these interactions, many have stated that, through the avenues discussed above, professional social media use has positive educational impacts and perceived as improving patient care. A survey in a medical school in Nigeria showed that almost 70% of the respondents are favorably disposed to pathology education *via* social media (21). In the last few years, a few international, predominantly survey-based studies have attempted to measure the impact of the use of social media on dissemination of knowledge in pathology. Results have been overwhelmingly positive in the perception of improved impact as measured through questionnaires and personal experiences; users affirm that teaching cases have improved their knowledge and practice of pathology. Teaching posts, like Tweetorials with embedded pre- and post-tutorial quizzes, have documented increasing learning based on higher percentages of correct answers after the educational post (1, 7, 14, 21, 57, 58). Social media analytics—tools embedded within Twitter—track the impact of a post by documenting impressions (potential viewers) and engagements (viewers that interact with the post). Good engagement rates have been defined (between 1–5%) (58). These metrics reflect the potential reach of the message/tweet and the extent of interaction it generates. They can be useful to provide feedback to content-creators regarding the quality and visibility of their posts. These same metrics inform how journals track the impact of their articles and academics illustrate the reach of a teacher/scholar's work (5, 14, 18). While we are not aware of a specific study to do so to date, social media analytics could feasibly represent a useful tool to help quantify pathology knowledge acquisition in Nigeria.

Potential challenges and limitations also exist for using social media in pathology. One of the most important concerns is protecting the privacy of patients. This is ensured by never including any protected health information such as patient name, age, sex, or date, and location of service. Clinical information should be minimized or altered (1, 5, 7, 18). Sharing of completely de-identified pathology images or other information for educational purposes on social media is legally and ethically acceptable even without patient permission. Legal concerns are understandable; however, no lawsuits based on misuse of social media by pathologists have been filed thus far (6, 7, 18).

Many cases posted on social media are intended mostly for academic purposes. However, pathologists working where there is limited access to ancillary testing or expert consultation services may share cases with diagnostic challenges for assistance. Selection bias and the possibility of only seeing a fraction of the material contained on the actual slide are potential disadvantages to providing opinions on cases shared as a few images on social media. Whole-slide imaging may soon obviate this potential problem but is not yet widely available. Ensuring the accuracy and reliability of medical opinions expressed on social media is also a challenge. However, these platforms open cases up for comments by hundreds of potential “peer reviewers” including experts in the particular field. The resultant constant fact-checking thereby effectively counters false information. Pathologists that share cases for discussion must realize that as the patient's physician, they hold sole responsibility for the final diagnosis and its impact on the patient's care regardless of what other colleagues and experts say. Hence, opinions *via* social media must be regarded as unofficial consultation (1, 7, 18).

Despite the potential benefits, wide reach and availability of these platforms, social media as a tool for advancement of pathology knowledge is still undersubscribed in Nigeria. Contributing factors include poor awareness, unsatisfactory and expensive internet services, and restriction of access to these services during periods of political unrest. Improving awareness of and support for these tools will ideally help mitigate some of the challenges faced practicing pathology in low and middle-income regions of the world.

## Data Availability Statement

The original contributions presented in the study are included in the article/supplementary material, further inquiries can be directed to the corresponding author/s.

## Author Contributions

OOF, KMI, and DAK contributed to the conception of the work. OOF and KMI wrote the first draft of the manuscript. OAO and DAK provided materials for the initial draft and wrote sections of the manuscript. All the authors contributed to the revision of the manuscript and approved the version submitted.

## Funding

Dartmouth College Provost / Library Fund to Support Open Access Publication.

## Conflict of Interest

The authors declare that the research was conducted in the absence of any commercial or financial relationships that could be construed as a potential conflict of interest.

## Publisher's Note

All claims expressed in this article are solely those of the authors and do not necessarily represent those of their affiliated organizations, or those of the publisher, the editors and the reviewers. Any product that may be evaluated in this article, or claim that may be made by its manufacturer, is not guaranteed or endorsed by the publisher.
