# Supplementary material for: The Influence of Social Media in Promoting Knowledge Acquisition and Pathology Excellence in Nigeria
Source: Front Med (Lausanne). 2022 Jun 3;9:906950. doi: 10.3389/fmed.2022.906950 (PMC9203859; doi:10.3389/fmed.2022.906950)
Supplement: Supplementary file 1 [file Table_1.docx]

APPENDIX

Supplementary Material

Examples of some Twitter handles that share excellent educational resources on Twitter that have been of great benefit

1. Group collaborations
   1. PathElective <https://www.pathelective.com/> Founded by
      Dr Kamran M. Mirza (@KMirza) and Cullen M. Lilley (@cullen_lilley) who are themselves avid Twitter users. The collaboration offers high-quality pathology elective material to those who do not have prior exposure to pathology rotations, especially medical students. The modules range from Anatomic Pathology, Clinical Pathology, and career advancement modules
   2. Virtual Pathology Grand Rounds @VirtualPathGR formed by a group of pathologists and sponsored by the American Society of Clinical Pathologists (ASCP) at the peak of the first wave of the COVID-19 pandemic to enable access to excellent educational materials free of charge.
   3. Pathcast: A pathology broadcast service founded by Dr. Emilio Madrigal (@EMadrigalDO) and Dr. Rifat Mannan (@MannanRifat03) in 2016. The channel streams live teaching sessions on YouTube and Facebook and encompasses live slide reviews and lectures.
2. Institutional Twitter handles with regular updates
   1. #DailyDx a hashtag describing daily pathology cases shared by the University of Michigan Department of Pathology (@UMichPath)
   2. Case of the Week (#COTW) by various pathology departments, including:
      1. Emory University (@EmoryPathology)
      2. Mount Sinai (@MountSinai_Path)
      3. Brigham and Women's Hospital (@BWHPath)
      4. University of Alabama at Birmingham (@UABPathology)
      5. University of Michigan (@UMichPath)
   3. Case of the Month by various pathology departments, associations and societies
      1. Department of Pathology and laboratory Medicine, University of Rochester Medical Centre (@Pathology_URMC)
      2. Georgia Association of Pathology (@GAPathologists
      3. The Pulmonary Pathology Society, PPS (@PulmPathSoc)
      4. College of American Pathologists (@Pathologists)
      5. International Society of Urological Pathology, ISUP (@IntSocUropath)
      6. Pathology Outlines (@Pathoutlines)
      7. The British Society for Oral and Maxillofacial Pathology, BSOMP (@theBSOMP)
3. Pathology Societies
   1. Texas Society of Pathologists (@TexPathol)
   2. American Society of Dermatopathology (@ASDPTweets)
   3. College of American Pathologists (CAP) (@Pathologists) provided a series of lectures also during the COVID-19 pandemic promoted with the hashtag #CAPvirtualPath
   4. Pathology Society of Philadephia (@philly_path), the oldest pathology in the US (founded in 1857)
4. Online Journal clubs
   1. Pathology Journal club (@Path_JC): A Journal club with a goal of stimulating discussion on research and articles at the forefront of the field of pathology. It was founded by a group of notable pathologists and avid Twitter users:
      1. Dr. Christina Arnold @CArnold_GI
      2. Dr. Michael Arnold @MArnold_PedPath
      3. Dr. Matt DeNicola @drmattdenicola
      4. Dr. Simon Chiosea @chioseasi
      5. Dr. Eve Crane @evemariecrane
      6. Dr. Maren Fuller @marenwhymd
      7. Dr. Jerad Gardner @JMGardnerMD
      8. Dr. Sara Jiang @Sara_Jiang
      9. Dr. Matthew Wasco @Gleason4plus5
      10. Dr. Sean Williamson @Williamson_SR
   2. DermpathJC (@dermpathJC): Foremost example of a subspecialty pathology journal club. It is the first dermatopathology online journal club; it was founded by Dr. Silvija Gottesman (@SGottesmanMD) with the aim of promoting discussion on cutting edge dermatopathlogy articles on Twitter.
5. Educational Pathology Tweet Award (@PathTweetAward): Founded by Dr. Sanjay Mukhopadhyay @smlungpathguy) and Dr. Amy Deeken (@AmyHDeekenMD with a goal of funding an award for the best educational tweets that are nominated with the hashtag #PathTweetAward and collated by a team of judges. One of the authors of this article (O.O.F.) received the first prize for the trainee category in 2019 as a resident and is currently part of the team of judges after serving as the lead judge for a year.
